# Maternal Overnutrition Induces Long-Term Cognitive Deficits across Several Generations

**DOI:** 10.3390/nu11010007

**Published:** 2018-12-20

**Authors:** Gitalee Sarker, Daria Peleg-Raibstein

**Affiliations:** Laboratory of Translational Nutrition Biology, The Swiss Federal Institute of Technology, ETH Zürich, Schorenstrasse 16, 8603 Schwerzenbach, Switzerland; gitalee-sarker@ethz.ch

**Keywords:** cognition, maternal, mice, amino acids, overnutrition, obesity

## Abstract

Ample evidence from epidemiological studies has linked maternal obesity with metabolic disorders such as obesity, cardiovascular disease, and diabetes in the next generation. Recently, it was also shown that maternal obesity has long-term effects on the progeny’s central nervous system. However, very little is known regarding how maternal overnutrition may affect, in particular, the cognitive abilities of the offspring. We reported that first-generation offspring exposed to a maternal high-fat diet (MHFD) displayed age-dependent cognitive deficits. These deficits were associated with attenuations of amino acid levels in the medial prefrontal cortex and the hippocampus regions of MHFD offspring. Here, we tested the hypothesis that MHFD in mice may induce long-term cognitive impairments and neurochemical dysfunctions in the second and third generations. We found that MHFD led to cognitive disabilities and an altered response to a noncompetitive receptor antagonist of the N-Methyl-D-aspartic acid (NMDA) receptor in adult MHFD offspring in both second and third generations in a sex-specific manner. Our results suggest that maternal overnutrition leads to an increased risk of developing obesity in subsequent generations as well as to cognitive impairments, affecting learning and memory processes in adulthood. Furthermore, MHFD exposure may facilitate pathological brain aging which is not a consequence of obesity. Our findings shed light on the long-term effects of maternal overnutrition on the development of the central nervous system and the underlying mechanisms which these traits relate to disease predisposition.

## 1. Introduction

The prevalence of obesity is increasing worldwide and has reached epidemic proportions posing a major problem for healthcare [1]. The last World Health Organization (WHO) report classified 1.9 billion adults with about 2.8 million dying yearly [2]. Obesity is associated with an increased incidence of comorbidities like dyslipidemia, hypertension, and type 2 diabetes mellitus (T2DM) [1]. A particular concern emerged in the past two decades, namely obesity during pregnancy [3,4,5,6]. Nearly two-thirds of women of childbearing age (19–44) are overweight, and 36.5% are obese. Therefore, it is not surprising that this epidemic also affects children of all ages with nearly 41 million children under the age of five years classified as overweight or obese [2]. It is already known that the obesity epidemic cannot be explained only as a result of an affluent lifestyle, reduced physical activity, or a genetic predisposition. Evidence has suggested that it may originate from environmental factors already present early in development during fetal life [7,8,9]. A maternal hyper-nutritional state, due to pre-existing T2DM, gestational diabetes or obesity, generates long-term risks of obesity and other diseases for the child as a consequence of either shared genes, environmental factors, or a combination of both [3,4,5,10]. Parents not only create food environments for their children but also influence their eating behaviors, taste preferences, and food choices [11,12,13,14]. The in utero environment, which profoundly affects the fetal developmental processes, is considered as important as genes or family habits in determining the predisposition to long-term health outcomes [8,9,15]. Recently, an association between maternal obesity and mental disorders including a cognitive decline in the progeny was indicated [10,16,17]. A handful of studies linked maternal obesity and unhealthy eating with cognitive abnormalities in the offspring (for review [17,18]). More specifically, maternal obesity was linked to a reduction in child IQ scores, cognitive test scores, and intellectual disability in the children. Additionally, other studies suggested that an association might exist between maternal obesity and increased symptoms of attention deficit hyperactivity disorder (ADHD) in children [19,20,21]. Although the association between maternal obesity and the offspring’s cognitive disabilities was described, the current literature in humans is still scarce and the findings are inconsistent [17]. The underlying mechanisms leading to a higher susceptibility of the progeny to develop cognitive abnormalities later in life is still unknown. Thus far, human studies fail to provide a causal link due to a variety of confounding factors, such as genetic predisposition, food preferences, socio-economic status, etc. Therefore, animal studies are invaluable in studying the causal relationship between maternal overnutrition and neuropsychiatric disorders in the offspring. Animal models of maternal overnutrition, induced by a high-fat diet (HFD) exposure, showed an increased risk of behaviors associated with mental health disorders in the offspring. We and others have shown that rodents exposed to a maternal high-fat diet (MHFD) during early development also show different behavioral alterations such as increased anxiety, changes in reward-related behaviors, and impaired cognitive function [22,23,24,25,26,27,28,29,30]. Additionally, in our animal model, we could show that MHFD exacerbates cognitive- and metabolic aging in the offspring that were associated with alterations in amino acid levels in the hippocampus and prefrontal cortex [31].

The current study was designed to further test the impact of maternal overnutrition on learning and memory performance in subsequent generations. We utilized our established mouse model of MHFD over a time period of nine weeks, prior to conception and during gestation and lactation, in different learning and memory paradigms. We were also interested in whether MHFD affected extinction learning of active avoidance conditioning. Extinction may be interpreted as a new type of association learning and does not represent unlearning of an old association [32,33]. Further, we also assessed locomotor activity in response to a systemic injection of a non-competitive N-Methyl-D-aspartic acid (NMDA) receptor antagonist, dizocilpine. Since learning and memory functions are known to be dependent on the integrity of the medial prefrontal cortex (mPFC) and the hippocampus, we measured excitatory and inhibitory neurotransmission in these brain regions.

## 2. Materials and Methods

### 2.1. Animals

C57BL/6 mice were obtained from Charles River, Sulzfeld, Germany at the age of 10 weeks. Animals were acclimatized for two weeks to the new environment, which was a temperature (21 ± 1 °C) and humidity (55 ± 5%) controlled facility under a reversed light-dark cycle (lights off: 7:00–19:00). Mice had ad libitum access to chow food [Kliba-Nafag 3430, Kaiseraugst, Switzerland; major nutrients: 18.5% crude protein, dry matter 88%, crude fat 4.5%, 54.2% nitrogen free extract (NFE)] and water. All mouse experiments described in the present study had been approved by the Zurich Cantonal Veterinary Office, Switzerland and were conducted in accordance with the Animal Welfare Ordinance (TSchV 455.1) of the Swiss Federal Food Safety and Veterinary Office.

### 2.2. Experimental Design

**Figure 1:**
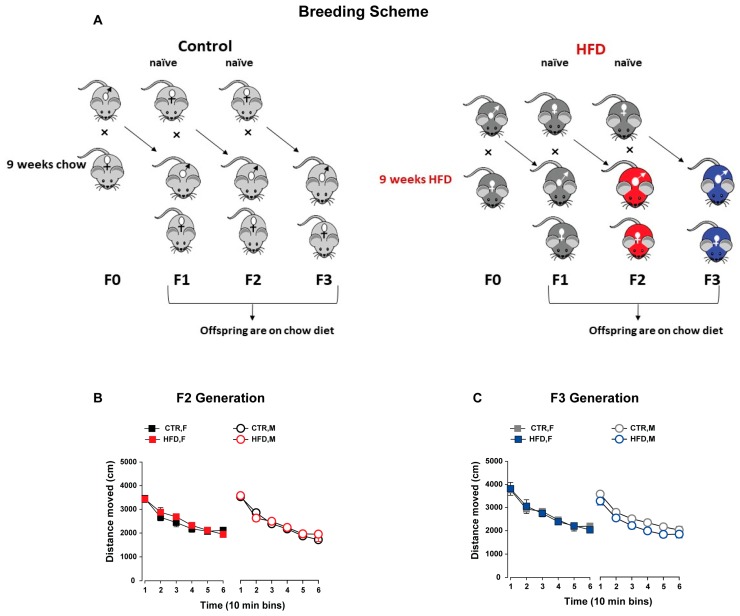
Breeding scheme and spontaneous locomotor activity. (**A**) Experimental design illustrating the breeding scheme. Female mice (F0) were fed either a (high-fat diet) HFD or chow diet for a total of nine weeks (three weeks prior to conception, three weeks during gestation, and three weeks during lactation) to obtain the F1 HFD and F1 control (CTR) offspring, respectively. F1-HFD and CTR males were mated with naïve females to generate the F2 offspring, respectively. The F2-HFD and CTR males were mated with naïve females to obtain the F3 offspring. All offspring from both groups were maintained on an ad libitum chow diet from weaning. Colors of the mice are matched with the color codes used for the groups in subsequent graphs. (**B**,**C**) General locomotor activity in the open field. The line plot shows the distance travelled (in cm) in successive 10-min bins for male and female offspring in the F2 (**B**) and F3 generation (**C**). All values are means ± SEM. N (F2 CTR) = (10 M, 10 F), N (F2 HFD) = (10 M, 10 F), N (F3 CTR) = (8 M, 8 F), N (F3 HFD) = (8 M, 8 F), HFD = high-fat diet, CTR = control diet, F = female, M = male.

F0 female mice were exposed to either a HFD (Kliba-Nafag 2127, Kaiseraugst, Switzerland; 60% energy from fat; major nutrients: 23.9% crude protein, dry matter 92%, crude fat 35%, 23.2% NFE) or normal laboratory chow diet (Kliba-Nafag 3430) for a total of nine weeks (three weeks before conception, three weeks during gestation, and three weeks during lactation) as described previously [23,34]. These diets were not matched for the micro- and macronutrients composition and concentrations. F0 male breeders were fed only a normal chow diet. F0 dams fed on HFD were neither obese during this whole period nor showed any abnormalities in maternal care during gestation and lactation [23]. Upon weaning on postnatal day (PND) 21, F1 offspring from both groups (HFD-fed dams and chow-fed dams) were given ad libitum access to a chow diet. To investigate the transgenerational effects, paternal lineage was followed in order to exclude the maternal confounding factors such as hormones, intrauterine milieu, milk composition, and maternal care [35,36]. In particular, transgenerational transmission via the maternal lineage likely relies on the complex maternal–fetal/neonatal interaction, whereas transmission through the paternal lineage suggests germ-line reprogramming [35,36]. To generate the F2 offspring, F1 males born to HFD and chow-fed dams were mated with naïve primiparous female mice reared on a chow diet. The males were removed from the mating cage once the vaginal copulation plug was confirmed. Pregnant dams from both groups were kept on a chow diet throughout gestation and lactation. Similarly, F3 offspring were generated by mating F2 males from HFD- and chow-fed ancestors with naïve primiparous females. The breeding scheme was shown in Figure 1A. One offspring per litter from multiple independent litters was selected for cognitive and neurochemical assessments to control for a litter effect [37]. To minimize the effects of prior behavior testing, behaviorally naïve offspring were allocated for each test. The groups were named after the ancestors (F0 dams) in both the F2 and F3 generations where offspring born to HFD-fed ancestors belong to the HFD group and offspring from chow-fed ancestors belong to the control diet (CTR) group. For each generation, the experiments and assessments started when offspring reached adulthood at PND 70. All behavioral experiments were carried out during the dark phase under red light or under dim light illumination that is stated for the specific paradigms below.

### 2.3. Open Field (Spontaneous Locomotor)

The spontaneous locomotor activity was assessed in the open field paradigm. The apparatus consisted of four identical (40 cm × 40 cm × 35 cm) gray opaque arenas. The test was conducted under dim lighting. A computerized tracking system (Noldus Technology, Wageningen, The Netherlands) was used to monitor activity. The locomotor activity was indexed by the distance travelled in cm in the entire arena throughout the 1 h testing period and expressed as a function of six successive 10 min bins.

### 2.4. Y-Maze (Working Memory)

The spatial working memory was tested in the Y-maze. The apparatus was made of three identical arms (50 cm × 9 cm; length × width) surrounded by 10-cm high transparent Plexiglas walls. The three arms radiated from a central triangle (8 cm on each side) and were spaced 120° from each other (Med Associates Inc., St Albans City, VT, USA). The apparatus was placed 90 cm above the floor in a testing room enriched with external spatial cues. The different activity variables were monitored by a computerized tracking system (Noldus Technology, Wageningen, The Netherlands). The time spent and distance moved in each of the three arms as well as in the center zone was calculated as previously described [38].

The working memory test consisted of two phases; the sample and test phases. In the sample phase, mice were allowed to freely explore two arms, the start arm and the familiar arm, respectively, for 5 min. The access to the remaining arm (the novel arm) was blocked by an opaque barrier. At the end of the sample phase, the animals were kept in a holding cage for the specific delay (10 min, 1 h, and 24 h) prior to the test phase. The allocation of the three arms to a specific spatial location was counterbalanced across subjects. The floor was properly cleaned after each trial to minimize the olfactory cues. During the test phase, mice were introduced to the maze again after a specific retention interval. In this phase, animals had access to all three arms and could freely explore the maze for 5 min. For each of the delays, a new cohort of experimentally naïve mice was used. The percentage of time spent in the novel arm during the test phase was measured by the formula [time spent in the novel arm/ (time spent in all arms)] × 100 and was indexed as the spatial working memory.

### 2.5. Two-Way Active Avoidance Learning

The apparatus consisted of four two-way shuttle boxes (Model H10-11M-SC, Coulbourn Instruments, Allentown, PA, USA) as fully described elsewhere [39]. The experiment included two phases; the acquisition and extinction phase. During the acquisition session, mice were placed in the box and were exposed to 100 avoidance trials which were administered at variable inter-trial interval (ITI) ITIs. A trial began with the onset of the conditioned stimulus (CS; tone). If the animal shuttled to the next chamber within 5 s of the CS onset, the CS was terminated and the mice avoided the electric shock on that trial (avoidance response). Avoidance failure immediately generated an electric foot shock of 2 s that could be ended by a shuttle response during this phase (escape response). The number of avoidance responses in successive blocks of 10 trials were expressed as percentage avoidance responses. This was used as an index of avoidance learning. During the extinction phase, the mice were placed in the same box and were exposed to 100 tone trials, but this time with a “no-shock condition”.

### 2.6. Prepulse Inhibition (PPI)

Sensory-motor gating was examined using the prepulse inhibition of an acoustic reflex paradigm. The apparatus consisted of four startle chambers (SR-LAB, San Diego Instruments, San Diego, CA, USA) as fully described elsewhere [40]. At the beginning of a trial, mice were placed inside the apparatus and acclimatized for 2 min with 65 dB background noise. After habituation, the animals were presented with 10 blocks of 16 discrete trials. Each block consisted of four different trial types: (1) three pulse alone trials (100, 110, 120 dB), (2) three prepulse alone trials (+6, +12, +18 dB above background), (3) nine prepulse-pulse trials and (4) a single no stimulus trial. PPI obtained at each prepulse intensity was indexed by percentage PPI (%PPI) calculated as %PPI = [1 − (prepulse-plus-pulse/pulse-alone)] × 100%.

### 2.7. Pharmacological Evaluation

#### 2.7.1. Locomotor Response to a Systemic Dizocilpine in the Open Field

The test was carried out in an open field (as described above). First, the mice were allowed to explore the open field arena for 30 min (baseline phase). The mice were then injected with a vehicle solution (0.9% NaCl solution) and immediately placed in the open field. Locomotor activity was measured for 30 min as described above. The mice were then removed from the apparatus and were administered dizocilpine (MK-801). They were immediately returned to the same arena, and the locomotor response to the acute drug challenge was monitored for a period of 120 min. Dizocilpine (Sigma–Aldrich, Schnelldorf, Germany) was dissolved in a sterile saline solution to achieve the desired concentration for injection via the intraperitoneal route at a volume of 5 mL/kg, 15 min before testing. The drug solution was freshly prepared on the day of testing. Locomotor activity levels were indexed by the distance moved in cm in the entire arena (expressed as a function of 10 min bins) throughout the testing period; 30 min (baseline phase), 30 min (saline phase), and 120 min (drug phase).

#### 2.7.2. Postmortem Neurochemical Evaluations

A naïve cohort of adult mice from both generations was employed for postmortem neurochemical assessments to exclude the possible effects of prior behavioral testing. At sacrifice, brains were rapidly extracted and 1 mm thick coronal sections were prepared using a sharp razor blade. The brain regions of interest such as the mPFC, dorsal, and ventral hippocampus were isolated according to the mouse brain atlas (K. Franklin and G. Paxinos). Tissue punches from the left and right hemispheres were combined, weighed, and placed in a 1.5 mL aliquot containing ice cold 300 μL 0.4M HClO4 and homogenized using ultrasound. After centrifugation at 10,000× *g* for 20 min at 4 °C, the clear supernatant layers were collected and filtered through a 0.2 μm nylon filter to separate the insoluble residue. This solution was immediately frozen and stored at −80 °C until further processing in the high-performance liquid chromatography (HPLC; Ultimate 3000, Thermo Scientific, Reinach, Switzerland) system. Levels of amino acids [gamma-aminobutyric acid (GABA), glutamate, aspartate, glycine, and taurine] were measured and analyzed using an electrochemical detector (ECD-3000RS, Thermo Scientific, Reinach, Switzerland) with a coulometric cell (6011RS, Thermo Scientific, Reinach, Switzerland). The brain tissue samples were mixed with the derivatizing reagent o-phthaldialdehyde (OPA) and 2-mercaptoethanol (βME) to perform the automated precolumn amino acid derivatization. After 2 min, the mixed samples were injected into the analytical system via a refrigerated autoinjector (Thermo Scientific, Reinach, Switzerland) equipped with a 100 μL injection loop. The samples were then separated on a reversed-phase column (4.6 × 80 mm, 3 µm Thermo Scientific, Reinach, Switzerland). The mobile phase consisted of monosodium phosphate, citric acid, ethylenediaminetetraacetic acid (EDTA), 10% methanol, 15% acetonitrile adjusted to pH of 5.6. An HPLC pump (ISO-3100BM, Thermo Scientific, Reinach, Switzerland) pumped the mobile phase throughout the system at a flow rate of 0.3 mL/L. Data acquisition and calculations were conducted using a chromatography workstation (Chromeleon, Thermo Scientific, Reinach, Switzerland).

#### 2.7.3. Statistical Analyses

All statistical analyses were conducted using the statistical software StatView (Abacus Corporation, Baltimore, MD, USA (version 5.0)). Data were analyzed using the analysis of variance (ANOVA) followed by post-hoc comparisons (Fisher’s least significant difference) or one-way ANOVA whenever appropriate. Spontaneous locomotor activity indexed by the distance moved was analyzed using a 2 × 2 × 6 (maternal exposure x sex x 10-min bins) repeated-measures ANOVA. The spatial working memory as indexed by the percentage of time spent in the novel arm during the test phase was subjected to a 2 × 2 (maternal exposure × sex) repeated-measures ANOVA. To analyze the avoidance response in the two-way active avoidance learning a 2 × 2 × 10 (maternal exposure × sex × 10-trial blocks) repeated-measures ANOVA was used. In the PPI test, %PPI was analyzed using a 2 × 2 × 3 × 3 (maternal exposure × sex × pulse × prepulse) repeated-measures ANOVA. Dizocilpine induced locomotor activity was subjected to a 2 × 2 × 12 (group × sex × 10-min bins) repeated-measures ANOVA. For the postmortem neurochemical analysis, each amino acid level was analyzed using a 2 × 2 × 3 (maternal exposure × sex × brain area) repeated-measures ANOVA.

## 3. Results

### 3.1. General Locomotor Activity

#### 3.1.1. F2 Generation

Offspring born to HFD ancestors did not show any difference in spontaneous locomotor activity compared to the CTR offspring (*F*_1,36_ = 0.277; *P* = 0.60, Figure 1B). ANOVA revealed a significant main effect of 10-min bins indicating a progressive reduction of locomotor activity over time which was similar in both groups (*F*_36,180_ = 102.07; *p* < 0.0001).

#### 3.1.2. F3 Generation

No difference in general locomotor activity was detected between the HFD and CTR offspring. Both offspring groups showed a gradual reduction in locomotor activity as was reflected by a significant main effect of 10-min bins (*F*_28,140_ = 124.97; *p* < 0.0001; Figure 1C). Female offspring showed higher locomotor activity than male offspring, as depicted by the main effect of sex (*F*_1,28_ = 5.86; *p* < 0.03).

### 3.2. Working Memory

#### 3.2.1. F2 Generation

**Figure 2:**
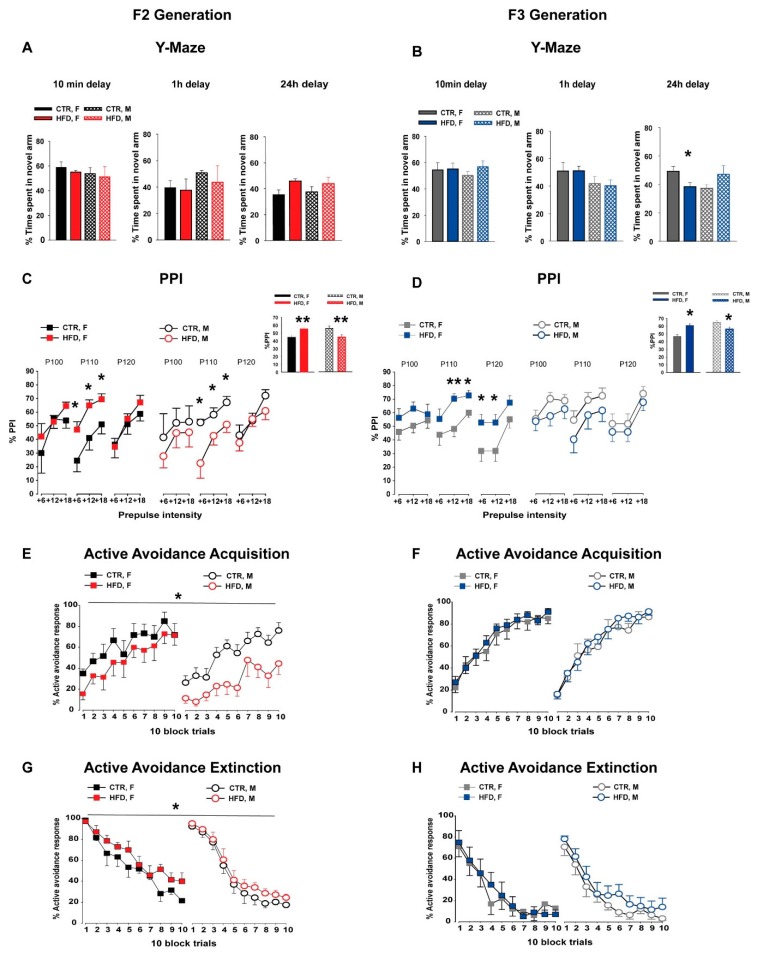
Cognitive assessments in the F2 and F3 generations. (**A**,**B**) Working memory in the Y-maze. The bar plot depicts the percentage time spent in the novel arm during the 5-min test phase for three different delays. The percentage time spent in the novel arm was assessed by the ratio of time spent in the novel arm compared to the total time spent in all three arms. Three separate animal groups were used for each of the delays. In the F2 generation (**A**), N = [10 min delay: CTR (4M, 4F), HFD (4M, 4F); 1 h delay: CTR (4M, 4F), HFD (4M, 4F); 24 h delay: CTR (4M, 4F), HFD (4M, 4F)]. In the F3 generation (**B**), N = [10 min delay: CTR (4M, 4F), HFD (4M, 4F); 1 h delay: CTR (4M, 4F), HFD (4M, 4F); 24 h delay: CTR (6M, 6F), HFD (5M, 5F)]. (**C**,**D**) Prepulse inhibition (PPI) of the acoustic startle reflex. The line graph depicts the %PPI as a function of three different pulses (100, 110, and 120 dB) and the corresponding prepulse intensities (+6, +12, and +18 dB above the background 65dB) in the F2 (**C**) and F3 (**D**) offspring. The bar plot in the inset shows the comparison of the mean %PPI between the groups. N (F2 CTR) = (8 M, 8 F), N (F2 HFD) = (8 M, 8 F), N (F3 CTR) = (9 M, 10 F), N (F3 HFD) = (10 M, 10 F). (**E**–**H**) Two-way active avoidance learning. The line plot shows the percent active avoidance responses in successive 10-trial blocks during the acquisition phase (**E**,**F**) and the extinction phase (**G**,**H**) in both generations. N (F2 CTR) = (6 M, 6 F), N (F2 HFD) = (6 M, 7 F), N (F3 CTR) = (10 M, 10 F), N (F3 HFD) = (10 M, 10 F). All values are means ± SEM. * *p* < 0.05; ** *p* < 0.001; *** *p* < 0.0001. AA = active avoidance, HFD = high-fat diet, CTR = control diet, F = female, M = male.

Offspring from both treatment groups spent more time in the novel arm compared to the other two arms in all delays, as reflected by a significant main effect of arms [10 min delay (*F*_2,24_ = 51.89; *p* < 0.001), 1 h delay (*F*_2,16_ = 3.89; *p* < 0.05), and 24 h delay (*F*_2,16_ = 4.49; *p* < 0.03)]. ANOVA revealed no significant main effect of group in the percentage of time spent in the novel arm following the 10 min (F_1,12_ = 0.36; *p* = 0.56), 1 h (*F*_1,8_ = 0.32; *p* = 0.59), and 24 h (*F*_1,8_ = 4.99; *p* = 0.06) delays, see Figure 2A. This suggests that MHFD exposure did not have any effect on spatial recognition memory in the F2 offspring. Neither the main effect of sex nor it’s interaction with group was observed in all three delays.

#### 3.2.2. F3 Generation

Both HFD and CTR offspring showed a clear preference for the novel arm during the three time intervals, as depicted by the main effect of arms [10 min delay (*F*_2,24_ = 68.80; *p* < 0.0001), 1 h delay (*F*_2,16_ = 11.29; *p* < 0.001), and 24 h delay (*F*_2,18_ = 4.49; *p* < 0.0001)]. ANOVA revealed no main effect of group in the percentage of time spent in the novel arm following the 10 min (*F*_1,12_ = 0.69; *p* = 0.42) and 1 h delays (*F*_1,8_ = 0.02; *p* = 0.89), indicating that there was no difference in spatial working memory between the groups following these test delays, see Figure 2B. Neither a sex effect nor its interaction with group was observed. In contrast, a significant group and sex interaction (*F*_1,18_ = 6.91; *p* < 0.02) was detected in percentage of time spent in the novel arm following a 24 h delay. Post hoc analysis further revealed that HFD-female offspring spent significantly less time in the novel arm (*p* < 0.05) during the test phase compared to the other offspring groups.

### 3.3. Prepulse Inhibition (PPI)

#### 3.3.1. F2 Generation

In the PPI test, %PPI was increased with the increased prepulse intensities in both groups as indexed by a main effect of prepulse intensities (*F*_1,36_ = 63.84; *p* < 0.0001, Figure 2C). ANOVA revealed a significant group × sex interaction (*F*_2,72_ = 5.52; *p* < 0.03) in %PPI. A subsequent post hoc analysis depicted that HFD-female offspring showed a significant enhancement of %PPI (*p* < 0.004), while HFD-male offspring showed a significant disruption of %PPI (*p* < 0.009) compared to the respective CTR offspring, see the inset of Figure 2C.

#### 3.3.2. F3 Generation

The level of prepulse inhibition was increased with increased levels of prepulse intensities in both groups, which was supported by a main effect of prepulse intensities (*F*_1,35_ = 61.05; *p* < 0.0001) and a significant pulse × prepulse intensities interaction (*F*_4,140_ = 10.81; *p* < 0.0001, Figure 2D). Similar to the F2 generation, a significant group × sex interaction was depicted (*F*_2,70_ = 5.93; *p* < 0.02). A follow-up post hoc confirmed a significant enhancement of %PPI in the HFD-female offspring (*p* < 0.0001), and a significant disruption of %PPI in HFD-male offspring (*p* < 0.02) as compared to the CTR-littermates, see the inset of Figure 2D.

### 3.4. Two-Way Active Avoidance Learning

#### 3.4.1. F2 Generation

During the acquisition phase, ANOVA of % avoidance responses yielded a main effect of block (*F*_9,189_ =23.89; *p* < 0.0001, Figure 2E), indicating that avoidance learning increased over time in both groups, which was measured by the increased number of avoidance responses. In addition, the analysis revealed a significant main effect of group (*F*_1,21_ = 6.81; *p* < 0.02), reflecting that HFD-F2 offspring displayed a reduced level of learning compared to the CTR-F2 offspring.

During the extinction phase, ANOVA of % avoidance responses yielded a main effect of block (*F*_9,189_ = 83.71; *p* < 0.0001), indicating that the proportion of avoidance responses decreased in both groups, see Figure 2G. HFD-F2 offspring showed reduced extinction compared to the CTR offspring. This observation was supported by a significant main effect of group (*F*_1,21_ = 4.63; *p* < 0.05). Furthermore, female offspring showed slower extinction compared to male offspring, see Figure 2G. This observation was supported by a significant main effect of sex (*F*_1,21_ = 21.74; *p* < 0.0001) and a significant sex × block interaction (*F*_9,189_ = 5.84; *p* < 0.0001).

#### 3.4.2. F3 Generation

The level of avoidance responses improved in both offspring groups during the acquisition phase over the 10-blocks, which was supported by a significant effect of block (*F*_9,324_ = 94.31; *p* < 0.0001; Figure 2F). Neither the main effect of group nor sex nor their interactions attained statistical significance. Similarly, no difference in avoidance responses during the extinction phase was observed between the groups, see Figure 2H.

### 3.5. Dizocilpine (MK-801) Sensitivity

#### 3.5.1. F2 Generation

**Figure 3:**
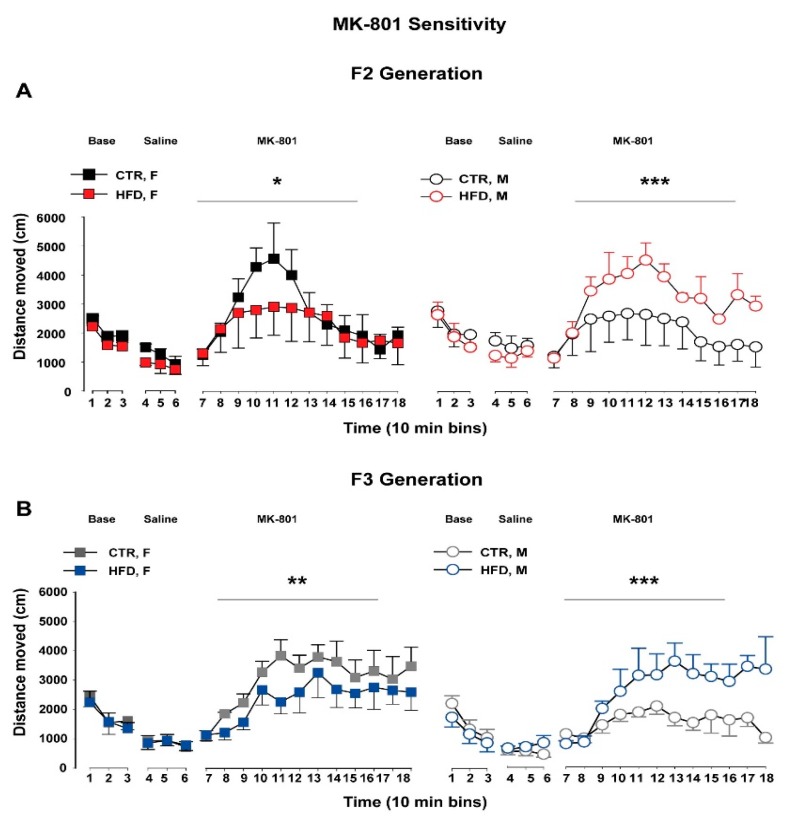
MK-801 sensitivity test in the F2 and F3 offspring. The line plot shows locomotor activity in the open field expressed as distance traveled (cm) per 10-min bin during baseline, following saline administration and following a systemic MK-801 injection in F2 (**A**) and F3 (**B**) offspring. N (F2 CTR) = (4 M, 4 F), N (F2 HFD) = (4 M, 4F), N (F3 CTR) = (5 M, 5 F), N (F3 HFD) = (4 M, 5F). All values are means ± SEM. * *p* < 0.05; ** *p* < 0.001; *** *p* < 0.0001. HFD = high-fat diet, CTR = control diet, F = female, M = male.

In the MK-801 sensitivity test, using a noncompetitive antagonist of the N-Methyl-D-aspartate receptor, no difference in baseline locomotor activity as well as the response following a saline injection was observed between the groups, see Figure 3A. Following an acute injection of MK-801, ANOVA revealed a significant group × sex × 10-min bins interaction (*F*_11,121_ = 2.02; *p* < 0.04). A subsequent post hoc analysis further confirmed that female HFD-F2 offspring showed a significant reduction of locomotor activity in response to the systemic MK-801 challenge compared to the CTR female littermates (*p* < 0.002). In contrast, male HFD-F2 offspring developed enhanced locomotor activity compared to the CTR-male offspring in response to the drug injection (*p* < 0.0005).

#### 3.5.2. F3 Generation

Offspring from both groups did not reveal any difference in spontaneous locomotor activity as well as in response to a saline injection, see Figure 3B. Following an MK-801 injection, ANOVA revealed a significant group × sex interaction (*F*_1,11_ = 7.39; *p* < 0.02) and a significant group × sex × 10-min bins interaction (*F*_11,154_ = 2.45; *p* < 0.008). A subsequent post hoc analysis confirmed that female HFD-F3 offspring developed significantly reduced locomotor activity in response to the MK-801 injection compared to the CTR-F3 littermates (*p* < 0.006). In contrast, male HFD-F3 offspring showed significantly increased locomotor activity compared to the CTR-F3 offspring following the MK-801 challenge (*p* < 0.0001).

### 3.6. Postmortem Neurochemical Brain Analysis

The basal levels of the excitatory (glutamate and aspartate) and the inhibitory (glycine, GABA, taurine) amino acids were measured and compared separately in the brain regions of interest (mPFC, dorsal hippocampus (d.Hippo), and ventral hippocampus (v.Hippo)) in both generations.

#### 3.6.1. Glutamate

**Figure 4:**
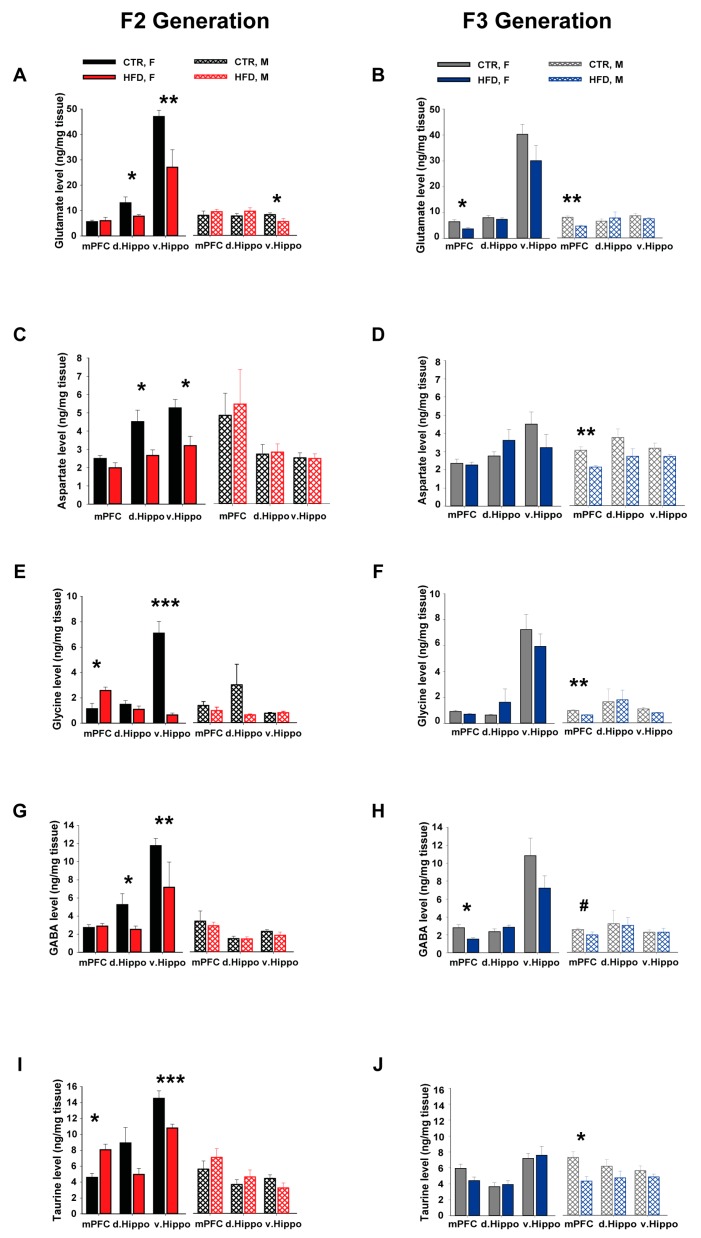
Postmortem neurochemical assessments in the F2 and F3 offspring. Levels of the excitatory amino acids glutamate (**A**,**B**) and aspartate (**C**,**D**), as well as the inhibitory amino acids including glycine (**E**,**F**), GABA (**G**,**H**), and taurine (**I**,**J**) were measured by postmortem HPLC in the F2 and F3 offspring. Amino acids were measured in the mPFC, d.Hippo, and v.Hippo and expressed as ng/mg tissue weight. N (F2 CTR) = (8 M, 8 F), N (F2 HFD) = (8 M, 9F), N (F3 CTR) = (8 M, 9F), N (F3 HFD) = (8 M, 8F). All values are means ± SEM. * *p* < 0.05; ** *p* < 0.001; *** *p* < 0.0001. mPFC = medial prefrontal cortex, d.Hippo = dorsal hippocampus, v.Hippo = ventral hippocampus, HFD = high fat diet, CTR = control, F = female, M = male.

In the F2 generation, we observed that HFD exposure did not affect glutamate levels in the mPFC (*F*_1,29_ = 0.10; *p* = 0.76; Figure 4A). In the d.Hippo, HFD-offspring showed altered levels of glutamate in a group- and sex-specific manner, which was supported by a significant group × sex interaction (*F*_2,64_ = 7.03; *p* < 0.02; Figure 4A). Subsequent post hoc analysis revealed that female HFD-F2 offspring had significantly lower levels of glutamate in the d.Hippo compared to the other groups (*p* < 0.03). In the v.Hippo, we observed HFD-induced alterations in glutamate levels which was supported by a significant main effect of group (*F*_1,34_ = 22.05; *p* < 0.0001; Figure 4A). Both male (*p* < 0.05) and female (*p* < 0.0003) HFD-F2 offspring showed significantly reduced glutamate levels in the v.Hippo compared to CTR offspring.

In the F3 generation, both male and female HFD-F3 offspring showed reduced levels of glutamate in the mPFC as compared to CTR-littermates which was supported by a significant main effect of group (*F*_1,25_ = 38.22; *p* < 0.0001; Figure 4B). No difference in glutamate levels was detected between F3-HFD and CTR offspring in the dorsal and ventral hippocampus, see Figure 4B.

#### 3.6.2. Aspartate

In the F2 generation, HFD-offspring did not show any difference in aspartate level in the mPFC compared to the CTR-offspring (*F*_1,29_ = 0.002; *p* = 0.96; Figure 4C). In the d.Hippo, ANOVA revealed a significant group × sex interaction (*F*_2,62_ = 4.41; *p* < 0.05; Figure 4C). Post hoc analysis confirmed that female HFD-F2 offspring had reduced levels of aspartate in the d.Hippo compared to the other groups (*p* < 0.03). In the v.Hippo, HFD-offspring showed altered levels of aspartate in a group- and sex-specific manner, as supported by a significant main effect of group (*F*_1,33_ = 5.23; *p* < 0.03), a main effect of sex (*F*_1,33_ = 20.98; *p* < 0.0001), and a significant group × sex interaction (*F*_2,66_ = 4.88; *p* < 0.04; Figure 4C). Subsequent post hoc analysis revealed that female HFD-F2 offspring showed significantly lower aspartate levels in this brain region compared to CTR-littermates (*p* < 0.02).

In the F3 generation, HFD offspring showed lower levels of aspartate in the mPFC compared to the CTR-offspring, which was supported by a significant main effect of group (*F*_1,28_ = 8.61; *p* < 0.007; Figure 4D). Subsequent post hoc analysis depicted reduced aspartate levels in male HFD-F3 offspring compared to the other groups (*p* < 0.0005). No difference in aspartate levels was observed between F3-HFD and -CTR offspring in the d.Hippo and v.Hippo, as shown in Figure 4D.

#### 3.6.3. Glycine

In the F2 generation, ANOVA revealed a significant group × sex interaction (*F*_2,58_ = 5.64; *p* < 0.03; Figure 4E) in the mPFC glycine levels. Post hoc analysis confirmed that female HFD-offspring had significantly higher levels of glycine in the mPFC compared to CTR-littermates (*p* < 0.03). In the v.Hippo, ANOVA yielded a significant main effect of group (*F*_1,25_ = 19.68; *p* < 0.0002), a main effect of sex (*F*_1,25_ = 18.44; *p* < 0.0002), and a significant group × sex interaction (*F*_2,50_ = 20.26; *p* < 0.0001), see Figure 4E. Post hoc analysis revealed a marked reduction of glycine levels in the HFD-female offspring compared to the other groups (*p* < 0.0001). No difference in the glycine levels was detected in the dorsal hippocampus between the groups.

In the F3 generation, HFD-F3 offspring showed significantly reduced levels of glycine in the mPFC compared to the CTR-littermates, which was supported by a significant main effect of group (*F*_1,27_ = 15.58; *p* < 0.0006; Figure 4F). The levels of glycine were lower in male HFD offspring compared to the other offspring groups, which was supported by a follow-up post hoc analysis (*p* < 0.03). HFD offspring did not show any difference in glycine levels in the dorsal and ventral hippocampus compared to CTR offspring, see Figure 4F.

#### 3.6.4. GABA

In the F2 generation, no difference in GABA levels was observed between the groups in the mPFC (group effect, *F*_1,29_ = 0.184; *p* = 0.67; Figure 4G). In the dorsal hippocampus, ANOVA revealed a significant effect of group (*F*_1,32_ = 4.34; *p* < 0.05), a main effect of sex (F_1,32_ = 9.31; P < 0.005), and a significant group × sex interaction (*F*_2,64_ = 4.54; *p* < 0.05; Figure 4G). Follow up post hoc analysis confirmed lower levels of GABA in female HFD-F2 offspring in the d.Hippo compared to the other groups (*p* < 0.04). In ventral hippocampus, HFD-offspring showed altered levels of GABA in a group and sex specific manner as supported by a significant main effect of group (*F*_1,34_ = 9.02; *p* < 0.006), a main effect of sex (*F*_1,34_ = 47.71; *p* < 0.0001) and a significant group × sex interaction (*F*_2,64_ = 7.31; *p* < 0.02; Figure 4G). Subsequent post hoc analysis revealed that female HFD-F2 offspring showed significantly reduced GABA levels in this brain region compared to CTR-littermates (*p* < 0.007).

In the F3 generation, HFD offspring showed lower levels of GABA in the mPFC compared to the CTR-offspring, which was supported by a significant main effect of group (*F*_1,28_ = 13.12; *p* < 0.002; Figure 4H). Subsequent post hoc analysis depicted reduced GABA levels in female HFD-F3 offspring compared to other groups (*p* < 0.006). No difference in GABA levels was detected between F3-HFD and –CTR offspring in d.Hippo and v.Hippo, see Figure 4D.

#### 3.6.5. Taurine

In the F2 generation, HFD-offspring showed higher taurine levels in the mPFC compared to the CTR-offspring, which was supported by a significant main effect of group (*F*_1,26_ = 6.84; *p* < 0.02; Figure 4I). In the v.Hippo, HFD offspring showed lower taurine levels in a group- and sex-specific manner, as supported by a main effect of group (*F*_1,26_ = 9.31; *p* < 0.006) and sex (*F*_1,26_ = 118.90; *p* < 0.0001). Follow-up post hoc analysis confirmed that female HFD-F2 offspring had lower taurine levels in the v.Hippo compared to the other groups (*p* < 0.0005). No difference in taurine levels was observed between the groups in the dorsal hippocampus.

In the F3 generation, ANOVA revealed a significant main effect of group (*F*_1,28_ = 15.39; *p* < 0.0006; Figure 4J), indicating that HFD offspring had lower levels of taurine in the mPFC compared to the CTR-offspring. Subsequent post hoc analysis showed that male HFD-F3 offspring had significantly lower taurine levels compared to other groups (*p* < 0.0005). No difference in taurine levels was observed between F3-HFD and CTR offspring in d.Hippo and v.Hippo, see Figure 4J.

## 4. Discussion

To the best of our knowledge, this is the first study to show that MHFD exposure leads to cognitive impairments across three generations. These cognitive deficits are mediated via the paternal lineage and are conserved up to the third generation. These offspring were never exposed to HFD throughout their life. This observation clearly indicates a true transgenerational inheritance [41,42]. The cognitive impairments were most evident in prepulse inhibition, as well as learning and memory in the two-way active avoidance paradigm. Further, MHFD led to pharmacological changes in response to MK-801 in the offspring. A significant sex-specific effect was observed in both F2 and F3 generations. Second- and third-generation male offspring showed %PPI disruption and enhanced sensitivity in response to MK-801. In contrast, female HFD offspring in both generations displayed facilitation of %PPI and reduced sensitivity to a systemic MK-801 challenge. The observed cognitive aberrations were further supported by sex-specific alterations of excitatory and inhibitory amino acids levels in the mPFC, ventral, and dorsal hippocampus of the HFD offspring compared to their controls in both generations, see Table 1.

The prevalence of obesity and neuropsychiatric disorders has accelerated dramatically over the last three decades [1,43]. An increasing body of evidence indicates that exposure to maternal obesity or overnutrition during critical periods of development adversely affects the offspring’s psychopathology [4,5,7,10,16,20,21,44,45,46]. Epidemiological studies have revealed a strong association between excessive gestational weight gain or unhealthy maternal diet and the increased predisposition to mental-health-related disorders including anxiety, depression, schizophrenia, and cognitive impairments [16,17,47]. Rodent research provides further evidence that MHFD exposure during gestation and lactation impairs the development of dopaminergic, serotonergic, and GABAergic neuronal pathways and leads to hyperactivity, enhanced anxiety-like behaviors, and diminished cognition in the adult offspring [22,25,26,27,48,49]. We showed in our previous study that MHFD exposure induced age-dependent impairments in learning and memory, as well as altered sensory motor gating function in first-generation offspring (F1) [31]. The cognitive dysfunction was paralleled with a significant reduction of excitatory (glutamate and aspartate) and inhibitory amino acids (GABA and taurine) in the ventral hippocampus and mPFC. While there is growing literature on the effects of MHFD on neuronal development and subsequent cognitive dysfunction in the offspring, very little is known regarding whether such effects are conserved across multiple descendants. In that respect, the present study is the only to report the persistent cognitive impairments together with altered brain function across three generations following a perinatal MHFD insult.

In the present study, we have employed a battery of experiments to evaluate cognitive functions such as various memory and learning paradigms, sensory motor gating functions, and pharmacological assessments. Second-generation-HFD offspring did not show deficits in spatial working memory in the Y-maze paradigm during the peripubertal (data not shown, [50]) and adult age, which is similar to the phenotypes observed in F1-HFD offspring [31]. The associative learning and memory measured in a two-way active avoidance paradigm were significantly impaired in F2-HFD offspring compared to their controls. The delay in learning and memory was not affected by locomotor activity since offspring did not differ in their spontaneous locomotor activity tested in the open field. Such associative memory impairments were also evident in the F1-HFD ancestors [31]. On the other hand, this phenotype was completely masked in the third-generation-HFD offspring. In the case of sensory-motor gating functions measured in the PPI paradigm, we observed a robust phenotype that was persistent across three generations. Notably, altered PPI response was stronger in the F2- and F3-HFD offspring compared to the phenotype observed in F1-HFD offspring. The different levels of phenotypic inheritance are aligned with other transgenerational models. We and several other groups have shown that the altered metabolic and behavioral traits induced by early-life environmental insults such as maternal overnutrition [34,51], stress [52], and infection [53] can be restricted to the direct descendant or mediated to the subsequent progeny while skipping some of the intermediate generations. While the actual cause of such generation-specific inheritance is not yet clear, it has been suggested that a differential degree of impact of environmental stimuli on the somatic and primordial germ cells may determine the transmission of traits and the emergence of phenotypes across generations [54].

In addition, complex sex-specific effects following a MHFD insult emerged in the F2 and F3 generations. In the PPI paradigm, female HFD offspring showed a potentiation of PPI similar to their ancestors while male HFD offspring showed a disruption of PPI. Similarly, a robust gender-specific effect was also depicted in MK-801 sensitivity in both generations. While female HFD offspring showed reduced locomotor response to MK-801, male-HFD offspring developed an enhanced sensitivity compared to control offspring. Similar sexual dimorphism in phenotypic inheritance is also evident in several other rodent models of early life stressors [55,56,57]. However, no studies to date have reported any definitive mechanism for such phenomena. Placental programming, sex hormones, the developmental plasticity of male and female gametes, as well as a sex-specific neurodevelopmental trajectory are some proposed factors that may contribute to such sexual segregations of phenotypes across generations [58,59,60].

An altered PPI response has been strongly associated with the pathophysiology of several neuropsychiatric disorders including schizophrenia, depression, and general anxiety disorders [61,62]. The disruption of PPI reflects the deficits in sensorimotor gating which underlie the sensory flooding, motor incoordination, and cognitive fragmentation. In rodent models, PPI has been shown to be disrupted by administration of dopamine agonists such as amphetamine and apomorphine, as well as activation of serotonergic receptors by several 5-hydroxytryptamine (5-HT) receptor agonists in the hippocampal-striatal-prefrontal cortical circuit [63,64,65]. In addition, a non-competitive NMDA-receptor antagonist such as dizocilpine (MK-801) has been reported to robustly disrupt PPI in rats and mice [66]. It has been proposed that the MK-801-mediated blockade of NMDA-receptors leads to excessive stimulation of non-NMDA receptors including α-amino-3-hydroxy-5-methylisoxazole-4-propionic acid (AMPA) and kainic acid (KA) receptors via inactivation of GABAergic interneurons [67,68]. The enhanced AMPA/KA receptor’s activity subsequently disinhibits glutamatergic neurotransmission, leading to disruption of PPI. Furthermore, prenatal exposure to MK-801 disrupts the parvalbumin GABAergic neuronal development in the mPFC, which leads to enhanced locomotor activity following MK-801 injection later in life [69]. On the other hand, potentiation of PPI has been strongly linked to reduced dopamine signaling in the striatum. Pharmacological manipulation with dopamine antagonists, such as major tranquilizers can induce an enhanced PPI response in rodents by inhibiting DA signaling [62]. In addition, neurodevelopmental manipulation with prenatal amphetamine insult has been shown to potentiate PPI in the adult offspring [70]. We have observed in the current study that F2- and F3-HFD offspring show a significant reduction in baseline glutamate and GABA levels in the mPFC and the hippocampal regions compared to control littermates. Interestingly, we reported in our previous study that F1, F2, and F3 offspring born to HFD-exposed dams also showed basal lower dopamine levels and increased D2 receptors expression in the ventral and dorsal striatum [23,34]. The observed neurochemical alterations, such as reduced dopamine, glutamate, and GABA levels in the striatum, mPFC, and hippocampus could indicate that altered dopaminergic, glutamatergic, and GABAergic signaling induced by MHFD might underlie the observed behavioral and pharmacological alterations in the HFD-offspring across generations. The structural changes and abnormal functional connectivity in these brain regions as a consequence of reduced glutamate and GABA levels are correlated with spatial and associative working memory deficits [71]. In addition, reduced aspartate, taurine, and glycine levels in the hippocampal and prefrontal regions of F2- and F3-HFD offspring, respectively, might partly contribute to the learning and memory deficits, as well as other cognitive abnormalities observed in the current study [72,73].

Obesity has a strong association with poor cognitive performance independent of associated medical co-morbidities. Obesity in rodents induced following chronic exposure to high-fat, high-sugar diets led to cognitive impairments, such as spatial memory and working memory deficits in the radial arm maze and Morris water maze, as well as contextual fear-conditioning impairments [74]. At the molecular level, fat-rich diets elevate pro-inflammatory cytokine levels, reduce hippocampal brain-derived neurotrophic factor (BDNF) levels, and disrupting the blood–brain barrier which promotes infiltration of blood-borne toxins in the hippocampus and other related brain regions [75,76,77]. Together, this may initiate neuroinflammation, synaptic remodeling, impaired neurogenesis, and neuronal apoptosis that affect cognitive function in obesity [78]. It is important to note that the F2 offspring born to HFD ancestors developed increased body weight, adiposity, insulin insensitivity, and altered lipid profiles [34]. In the F3 generation, the obesogenic- and metabolic-syndrome-like features were only evident in male HFD-offspring [34]. This may suggest that the cognitive impairments observed in F2- and F3-HFD offspring might be a consequence of an increased predisposition to obesity.

Our findings in this study demonstrate that MHFD exposure results in the transmission of cognitive deficits across three generations. The cognitive impairments are paralleled with altered amino acids in the mPFC, d.Hippo, and v.Hippo that are known to regulate these cognitive functions. We reported in our earlier study that F2 and F3 offspring born to HFD ancestors also showed lower dopamine levels in these brain regions [33]. Furthermore, F2- and F3-HFD offspring developed increased adiposity that might partly explain the observed phenotypes [33]. This data provides new perspectives on nutritional factors that may contribute to an individuals’ vulnerability to develop diseases later in life. The transmission of altered behavioral and molecular traits up to three generations via the paternal lineage strongly suggests the potential role of epigenetic modifications in the male germline for such inheritance. However, we have already shown that sperm methylome has little contribution for the transgenerational transmission of altered metabolic and addictive-like phenotypes induced by a MHFD insult due to the incomplete penetrance of altered DMR across generations [34]. Recently, the role of sperm small non-coding RNAs (sncRNAs) for such transmission of phenotypes has been reported in several rodent models [79,80,81]. The injection of a specific subpopulation of sperm small non-coding RNA in the embryo has been shown to recapitulate the programmed phenotype in the resultant offspring. The possible role of sperm sncRNAs may provide further insights into the underpinning molecular mechanism of inheritance of cognitive impairments induced by maternal overnutrition.

## Figures and Tables

**Table 1:** Summary of the observed cognitive phenotypes in F2 and F3 offspring.

Assessments	F1		F2		F3	
	Male	Female	Male	Female	Male	Female
General locomotor activity	ø	ø	ø	ø	ø	ø
Working memory	ø	ø	ø	ø	ø	↓ at 24 h delay
Prepulse inhibition	↑	↑	↓↓	↑↑	↓↓	↑↑
Active avoidance learning	↓	↓	↓	↓	ø	ø
MK-801 sensitivity	↑	↑	↑↑↑	↓	↑↑↑	↓↓

The table summarizes the observed behavioral phenotypes of F1, F2, and F3 offspring born to HFD and chow-fed dams. ø = no change; ↑ = increased relative to control, ↓ = decreased relative to control.
